# Long-term prognosis of patients with non-ST-segment elevation myocardial infarction according to coronary arteries atherosclerosis extent on coronary angiography: a historical cohort study

**DOI:** 10.1186/s12872-017-0710-3

**Published:** 2017-11-16

**Authors:** Karam Sadoon Alzuhairi, Peter Søgaard, Jan Ravkilde, Aziza Azimi, Michael Mæng, Lisette Okkels Jensen, Christian Torp-Pedersen

**Affiliations:** 1 Department of Cardiology, Aalborg University Hospital, Hobrovej 18, –9000 Aalborg, DK Denmark; 2 Department of Clinical Medicine, Aalborg University, Aalborg, Denmark; 3 Department of Health, Science and Technology, Aalborg University, Aalborg, Denmark; 4 Department of Cardiology, Aarhus University Hospital, Aarhus, Denmark; 5 Department of Cardiology, Odense University Hospital, Odense, Denmark

**Keywords:** Acute coronary syndrome, Myocardial infarction, Prognosis, Non-obstructive coronary artery disease

## Abstract

**Background:**

Patients with non-ST-segment elevation myocardial infarction (NSTEMI) without obstructive coronary artery disease (CAD) are often managed differently than those with obstructive CAD, therefore we aimed in this study to examine the long-term prognosis of patients with NSTEMI according to the degree of CAD on coronary angiography (CAG).

**Methods:**

We examined 8.889 consecutive patients admitted for first time NSTEMI during 2000–2011, to whom CAG was performed. Patients were classified by CAG into: 0-vessel disease (0VD), diffuse atherosclerosis (DA) (0% < stenosis <50%), 1-vessel disease (1VD), 2VD, and 3VD with stenosis ≥50%. Follow-up period: 13 years (median 4.5).

**Results:**

One-year mortality for NSTEMI patients with 0VD was 3.7%, DA 5.7%, 1VD 2.5%, 2VD 4.8%, and 3VD 11.5%. Non-diabetic 0VD patients had higher risk of mortality than 1VD patients (HR:1.59; 95% CI:1.21–2.02; *P* < 0.001), while those with diabetes mellitus (DM) had not significantly different risk. In addition 0VD group had higher risk of heart failure (HF) (HR 1.61; 95% CI: 1.39–1.88; *P* < 0.001), and lower risk of recurrent MI (HR:0.55; 95% CI:0.39–0.77; *P* < 0.001) compared with 1VD. For patients with DA; mortality and HF risks were higher than 1VD and not different than 2VD, while recurrent MI risk was not different than 1VD and lower than 2VD.

Finally, the DA group had higher risk of mortality if they had DM, higher risk of recurrent MI, and not different risk of HF and stroke compared with the 0VD group patients.

**Conclusion:**

Patients with NSTEMI and non-obstructive CAD (both normal coronaries and diffuse atherosclerosis) have a comparable prognosis to patients with one- or two-vessel disease. Patients with diffuse atherosclerosis have worse prognosis than those with angiographically normal coronary arteries.

**Electronic supplementary material:**

The online version of this article (10.1186/s12872-017-0710-3) contains supplementary material, which is available to authorized users.

## Background

Non-ST-segment elevation myocardial infarction (NSTEMI) with non-obstructive coronary arteries proven with coronary angiography is an important subgroup of patients with myocardial infarction (MI), because they are often managed differently, being less likely to receive recommended medical treatment after MI and more likely to discontinue double platelet inhibitors, than patients with NSTEMI with obstructive CAD [1, 2].

Studies investigating this subgroup reported a wide range of prevalence (4–13%) according to the definitions used, the type of MI, and the use of cardiac troponin (MI or acute coronary syndrome (ACS)) [3–5]. Previous studies suggested that factors predicting non-obstructive coronary arteries in MI patients are female, younger age, and lack of smoking and diabetes mellitus (DM) [2, 6, 7].

Previous studies showed that patients with NSTEMI with non-obstructive CAD had better prognosis compared with obstructive CAD [5, 6, 8–10]. Other studies reported a substantial risk in non-obstructive group with higher all-cause mortality [11] or similar risk of combined death, MI, admission for ACS, and non-fatal stroke compared with obstructive CAD group [2]. However, many of these studies were limited by a small sample size [1, 12] or a short duration of follow-up [6]. In addition, most of these prognostic studies compared patients with obstructive versus non-obstructive CAD, and studies dividing both these groups into subgroups according to coronary pathology are few [2, 10].

Therefore, the aim of this study was to assess the prognosis in a large number of NSTEMI patients divided into five groups according to their coronary artery atherosclerosis extent on the coronary angiography with a long-term follow-up.

## Methods

### Study design

This was a historical prospective study based on data collected from several Danish registries mentioned bellow. All citizens in Denmark have a unique identification number, which facilitates linkage between different registries on person-level.

### The registries

Since 1977 the Danish National Patient Register has collected data including discharge diagnosis from all admissions to Danish hospitals [13]. MI diagnosis is with high sensitivity and specificity [14, 15].

The Western Denmark Heart Registry has collected patient and procedure data since 1999 for all interventions in the hospitals in western Denmark; and it is a validated research source [16].

### Study population

We identified all patients discharged with first time NSTEMI or unspecific MI (ICD-10 codes: DI21.4 and DI21.9, respectively) in the Patient Register during the period January 1st, 2000 to August 31st, 2011, who underwent coronary angiography within 30 days. Patients discharged with unspecific MI diagnosis were included if NSTEMI diagnosis was confirmed from the Western Heart Registry, because this registry does not allow the use of (unspecific MI) diagnosis. From this registry clinical data and angiographic description of coronary arteries stenosis were obtained Patients were divided accordingly into five subgroups: zero-vessel disease (0VD) = angiographically normal coronary arteries; diffuse atherosclerosis (DA) = moderate focal or diffuse atherosclerosis either without stenosis ≥50%; one-vessel disease (1VD) with stenosis ≥50%; two-vessel disease (2VD) with stenosis ≥50%; or three-vessel disease (3VD) with stenosis ≥50%. Patients with left main stenosis ≥50% were included either in the 3VD group, if the right coronary was hypoplastic or with stenosis ≥50%, or in the 2VD group if the right coronary was without significant stenosis. From the Civil Registration System we obtained gender, age, and mortality status.

### Study outcomes

Recurrent MI was identified from the Patient Register using ICD-10 code: DI21 (all types of MI). To avoid misclassification due to transfer between hospitals, we made a program to merge related admissions into one, and added 5 days after the discharge day, where no recurrent MI can be considered.

Moreover, we did a sensitivity analysis where no recurrent MI was considered within the first 30 days. Stroke event defined using ICD-10 code: DI61(intracerebral haemorrhage), DI62 (other non-traumatic intracranial haemorrhage), DI63 (cerebral infarction), or DI64 (stroke, not specified). Patients with stroke diagnosis before NSTEMI were not included in stroke outcome analysis. To identify heart failure (HF) event, we used either ICD-10 code: DI50.9 (heart failure, unspecified) or DI25.5 (ischemic cardiomyopathy).

### Exclusion criteria

1. Missing data on coronary atherosclerosis description (628 (6%) of study population).
2. Previous MI.
3. Known with HF.
4. Prior revascularization treatment.

### Follow up and end points

During a median follow up period of 4.5 years (1.3–13 years) the outcomes: mortality, recurrent MI, HF, and stroke were registered. Follow-up started on the day of admission with first NSTEMI and ended on the 31st December 2012, or the date of emigration or death.

### Statistical analysis

Categorical variables are presented as numbers and percentages and compared using Chi-square test, while continuous variables presented as median with inter-quartile range, and compared using analysis of variance. Time to event curve was generated using Aalen-Nelson cumulative incidence estimator taking into account death as a competing risk.

Cox proportional hazard models were used to estimate hazard ratio with 95% confidence interval. The model was adjusted for: age, sex, DM, hypertension, current smoker status, renal insufficiency (defined as estimated glomerular filtration rate (eGFR) <60 ml/min/1.73m^2^ using MDRD equation), and overweight (defined as body mass index ≥25). Left ventricular ejection fraction (EF) was not included in the primary analysis because only 45% of patients had available measurements; however, an additional analysis was done separately for this subgroup. Model assumptions for proportional hazard and linearity were found valid. Effect modification was tested using likelihood ratio test for clinically relevant variables: age, sex, hypertension, DM, renal insufficiency, and smoking. There was a significant and clinically important effect modification of DM (*P* 0.003) on mortality outcome; therefore we did the analysis with and without DM. No significant effect modification was found for DM on the other outcomes or for the other variables on all outcomes.

For more details in statistical analysis, please see online appendix A. All statistical analyses were performed using the SAS statistical software V.9.2 (SAS Institute Inc., Cary, North Carolina, USA), and R version 3.02 (R Development Core Team).

## Results

Of 8889 first time NSTEMI patients who underwent coronary angiography, 1290 (14.5%) had non-obstructive coronary arteries. Of these 1290 patients, 988 (76.5%) had 0VD, and 302 (23.5%) had DA with no stenosis ≥50%. The proportion of patients with non-obstructive CAD increased throughout the study period reaching 18% in 2011.

Demographic data of the study population are showed in Table 1. Patients with 0VD had a comparable risk profile to those with 1VD except that the majority were females (59.9% vs. 29.6% *P* < 0.001), and they were less likely to be current smoker (32.4% vs. 42.7, *P* < 0.001) or overweight (57.7 vs. 67.3, *P* < 0.001). However, patients with 0VD were younger, more likely to be females, and less likely to have hypertension and DM than patients with DA. The DA group had similar characteristics to the 2VD group, except it included more women (44.0% vs. 23.3%, *P* < 0.001) and less overweight (55.6% vs. 66.7, *P* < 0.001). Patients with all sub-groups of obstructive CAD were significantly more frequently treated with revascularization (either percutaneous coronary intervention or coronary by-pass grafting (Table 1), they were also more likely to receive double anti-platelet therapy than patients with 0VD or DA (Table 1).

**Table 1:** Baseline characteristics of the study population

Variables	0VD (*N* = 988)	DA (*N* = 302)	1VD (*N* = 3295)	2VD (*N* = 2114)	3VD (N2190)	*P* values
Age (years)	62 {53, 72}	66 {56, 74}	63 {54, 71}	67 {59, 75}	71 {63, 78}	< 0.001
Female gender	585 (59.9)	131 (44.0)	966 (29.6)	489 (23.3)	587 (27.0)	< 0.001
Hypertension	368 (39.2)	143 (49.7)	1290 (41.6)	875 (44.4)	1088 (53.6)	< 0.001
Hyperlipidemia	427 (45.5)	144 (49.5)	1517 (48.9)	1060 (53.6)	1093 (53.9)	< 0.001
Diabetes mellitus	106 (11.0)	51 (17.2)	413 (12.9)	342 (16.6)	488 (23.0)	< 0.001
IHD in the family	347 (37.4)	113 (40.1)	1231 (40.5)	765 (39.3)	750 (37.9)	0.3072
Current smoker	293 (32.4)	100 (36.6)	1303 (42.7)	772 (39.4)	654 (33.0)	< 0.001
Overweight^a^	463 (57.7)	143 (55.6)	1764 (67.3)	1097 (66.7)	1059 (64.4)	< 0.001
Renal insufficiency^b^	107 (13.8)	39 (15.4)	353 (13.8)	310 (19.0)	457 (28.0)	< 0.001
EF < 50%	107 (22.1)	37 (25.2)	282 (19.5)	258 (28.7)	427 (42.3)	< 0.001
Previous stroke	36 (3.6)	15 (5.0)	113 (3.4)	111 (5.3)	189 (8.6)	< 0.001
Treatment						
Any revascularisation^c^	26 (2.6)	15 (5.0)	2764 (83.9)	1816 (85.9)	1688 (77.1)	< 0.001
PCI	21 (2.1)	11 (3.7)	2728 (82.8)	1588 (75.1)	769 (35.1)	< 0.001
CABG	5 (0.5)	4 (1.3)	36 (1.1)	228 (10.8)	919 (42.0)	< 0.001
Aspirin	883 (90.9)	288 (95.7)	3177 (96.6)	2039 (96.5)	2048 (93.8)	<0.001
P2Y12 receptor inhibitor	668 (67.7)	235 (78.1)	3097 (94.2)	1920 (90.9)	1642 (75.2)	<0.001
Beta blocker	758 (78.1)	263 (87.4)	2964 (90.1)	1892 (89.6)	1975 (90.5)	<0.001
ACE-inhibitor	428 (44.1)	147 (48.8)	1641 (49.9)	1175 (55.6)	1363 (62.4)	<0.001
Statin	808 (83.2)	277 (92.0)	3103 (94.3)	1968 (93.2)	1978 (90.6)	<0.001

Parameters presented as numbers (percentages from non-missing data) or median (25th, 75th percentile)

*Abbreviations*: *0VD* zero-vessel disease, *DA* diffuse atherosclerosis, *1VD* one-vessel disease, *2VD* two-vessel disease, *3VD* three-vessel disease, *IHD* ischemic heart disease, *EF* ejection fraction, *PCI* percutaneous coronary intervention, *CABG* coronary by-pass graft operation, *P2Y12-inhibitor* P2Y12 receptor inhibitor, *ACE* angiotensin-converting-enzyme

^a^Overweight defined as body mass index ≥25

^b^Renal insufficiency defined as estimated glomerular filtration rate < 60 ml/min/1.73m^2^ using MDRD equation

^c^Revasculrisation defined as PCI within 30 days, and CABG within 60 days of non-ST-elevation myocardial infarction

### Long-term prognosis of NSTEMI patients according to their coronary artery pathology

#### Mortality

**Fig. 1:**
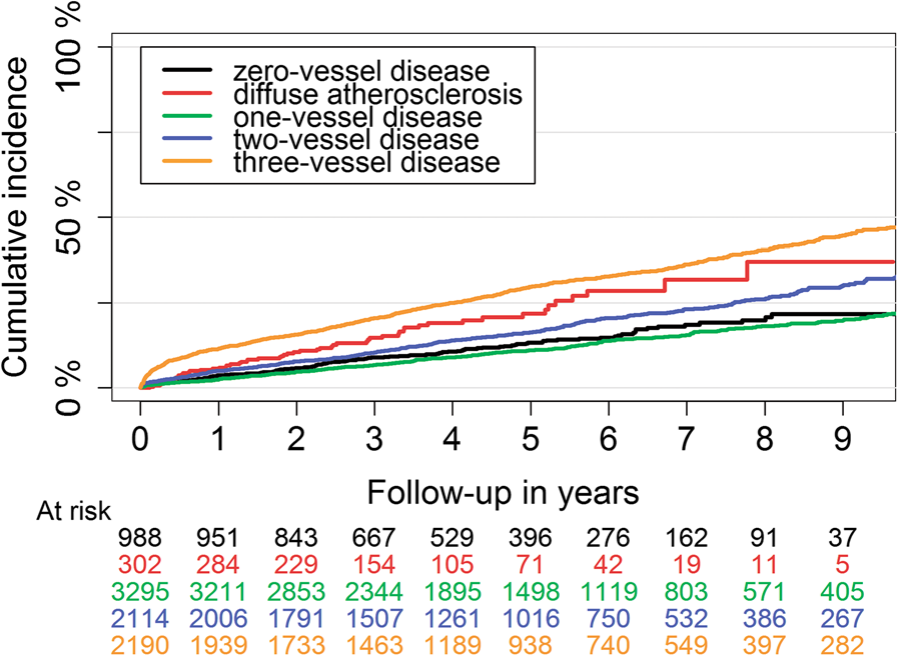
Long-term mortality in patients with non-ST-segment elevation myocardial infarction according to their coronary artery atherosclerosis extent

Dividing NSTEMI patients according to coronary artery disease extent revealed that one-year mortality for patients with 0VD, DA, 1VD, 2VD, and 3VD were 36 (3.6%), 17(5.6%), 80(2.5%), 105(5.0%), and 251(11.5%), respectively (Table 2). 1VD had the lowest and 3VD had the highest unadjusted cumulative mortality rate (Fig. 1).

**Table 2:** One-year and five-year prognosis of patients with NSTEMI according to their coronary artery atherosclerosis extent

Outcomes	0VD (*N* = 988)	DA (*N* = 302)	1VD (*N* = 3295)	2VD (*N* = 2114)	3VD (*N* = 2190)
1-year					
Death	36 (3.6%)	17 (5.6%)	80 (2.4%)	105 (5.0%)	251 (11.5%)
Recurrent MI	35 (3.5%)	19 (6.3%)	273 (8.3%)	285 (13.5%)	369 (16.8%)
Heart Failure	101 (10.2%)	40 (13.2%)	263 (8.0%)	249 (11.8%)	433 (19.8%)
Stroke	17 (1.7%)	4 (1.3%)	42 (1.3%)	37 (1.8%)	69 (3.2%)
5-years					
Death	120 (12.1%)	55 (18.2%)	327 (9.9%)	315 (14.9%)	609 (27.8%)
Recurrent MI	56 (5.7%)	29 (9.6%)	353 (10.7%)	360 (17.0%)	464 (21.2%)
Heart Failure	139 (14.1%)	45 (14.9%)	352 (10.7%)	367 (17.4%)	623 (28.4%)
Stroke	41 (4.1%)	16 (5.3%)	117 (3.6%)	94 (4.4%)	140 (6.4%)

The results presented in numbers (percent)

*Abbreviations*: *0VD* zero-vessel disease, *DA* diffuse atherosclerosis, *1VD* one-vessel disease, *2VD* two-vessel disease, *3VD* three-vessel disease

#### Patients with NSTEMI and without DM

**Fig. 2:**
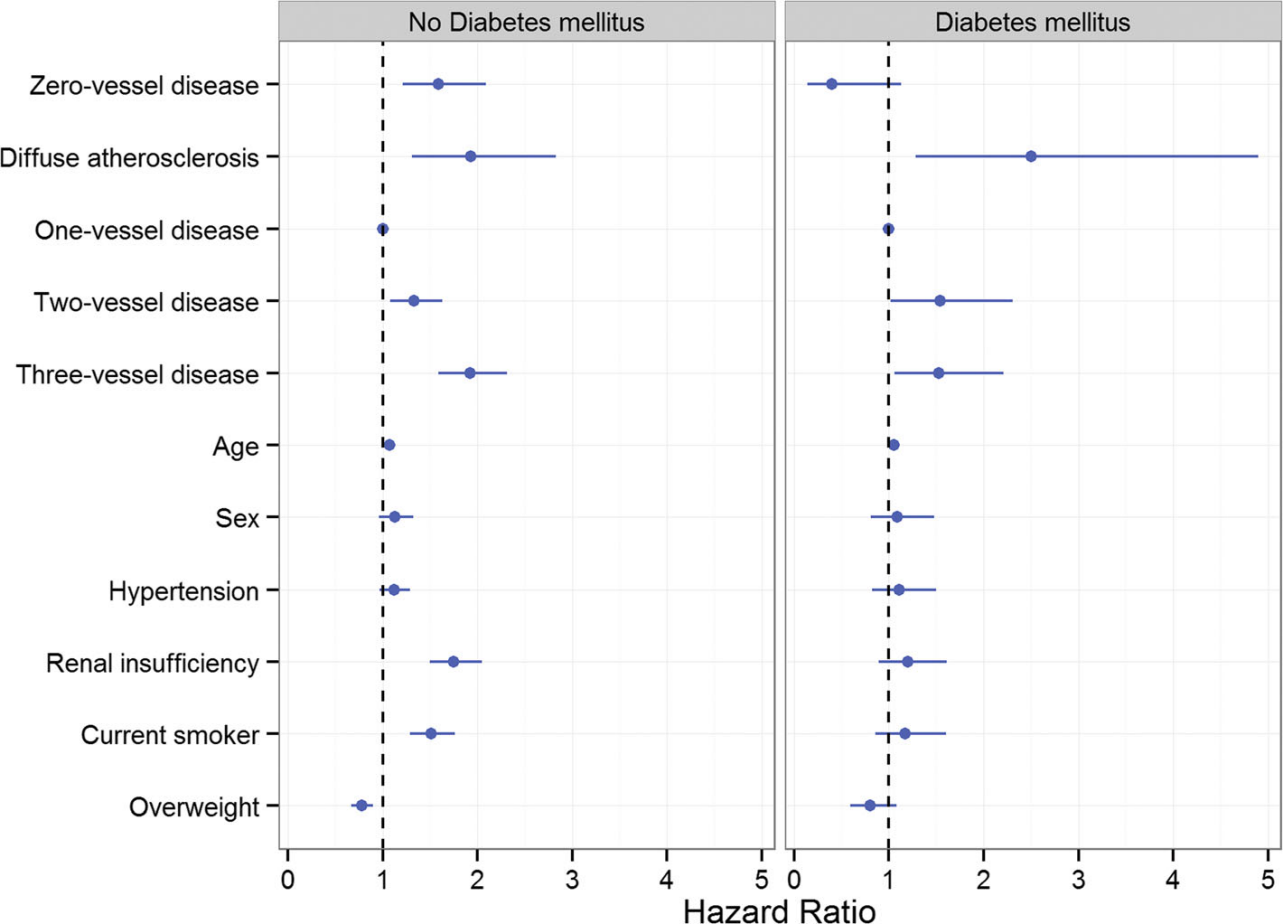
Adjusted mortality hazard ratio for NSTEMI patients according to their coronary artery pathology. 1VD group was used as a reference group. The model was adjusted for age, sex, hypertension, renal insufficiency (eGFR < 60 ml/min/1.73m^2^), current smoker status, and overweight (BMI ≥ 25)

After adjustment for covariates, mortality risk for the 0VD group was higher than the 1VD group (HR:1.59; 95% CI:1.21–2.02, *P* < 0.001) (Fig. 2, Additional file 1), and not significantly different from the DA (HR:0.82; 95% CI:0.54–1.26, *P* = 0.37), 2VD (HR:1.19; 95% CI:0.91–1.57, *P* = 0.21), and the 3VD groups (HR:0.83;95% CI:0.64–1.08*, P* = 0.17). On the other hand, patients with DA had higher risk of mortality compared with 1VD patients (HR:1.93; 95% CI:1.31–2.83, *P* < 0.001), nominally higher, but not statistically significant, compared with 2VD group (HR:1.44; 95% CI:0.97–2.13, *P =* 0.06), and not different than the 3VD group.

#### Patients with NSTEMI and DM

After multi-factorial adjustment, mortality risk for 0VD patients was not significantly lower than the 1VD group (Additional file 1), but significantly lower than the DA (HR:0.16; 95% CI:0.05–0.51, *P =* 0.002), 2VD (HR:0.26; 95% CI:0.09–0.72, *P* = 0.01), and the 3VD groups (HR:0.26; 95% CI:0.10–0.72, *P* = 0.009). For patients with DA and diabetes, mortality risk was higher than 1VD (HR: 2.5; 95% CI:1.28–4.90, *P* = 0.007), but not significantly different compared with both 2VD and 3VD.

#### Recurrent MI

**Fig. 3:**
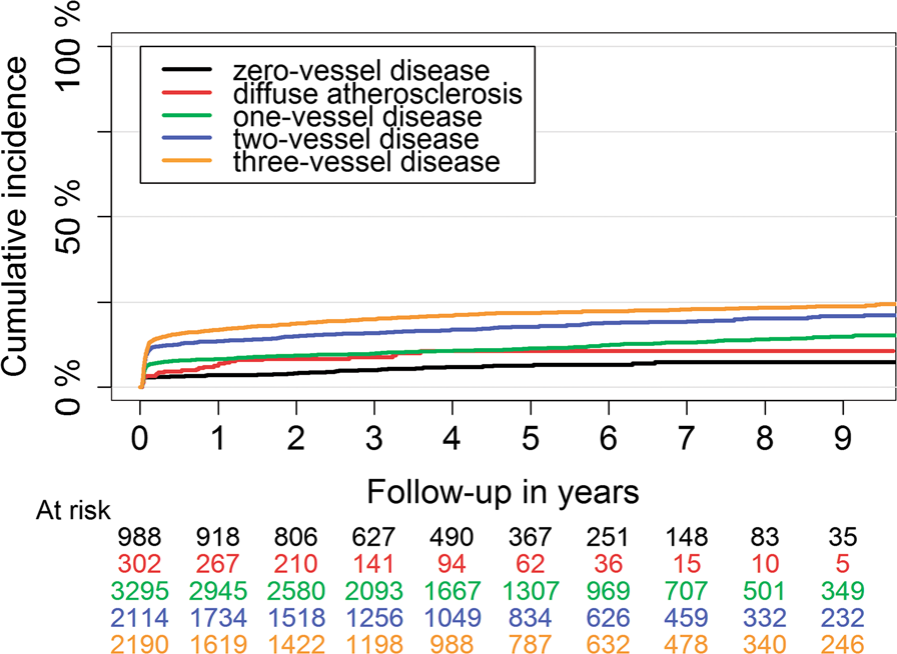
Long-term recurrent myocardial infarction cumulative incidence in patients with first NSTEMI divided by their coronary artery atherosclerosis extent

One-year risk of recurrent MI was lowest in patients with non-obstructive CAD both the 0VD and the DA groups (Table 2). The 3VD group had the highest incidence of recurrent MI throughout the study period (Fig. 3).

**Fig. 4:**
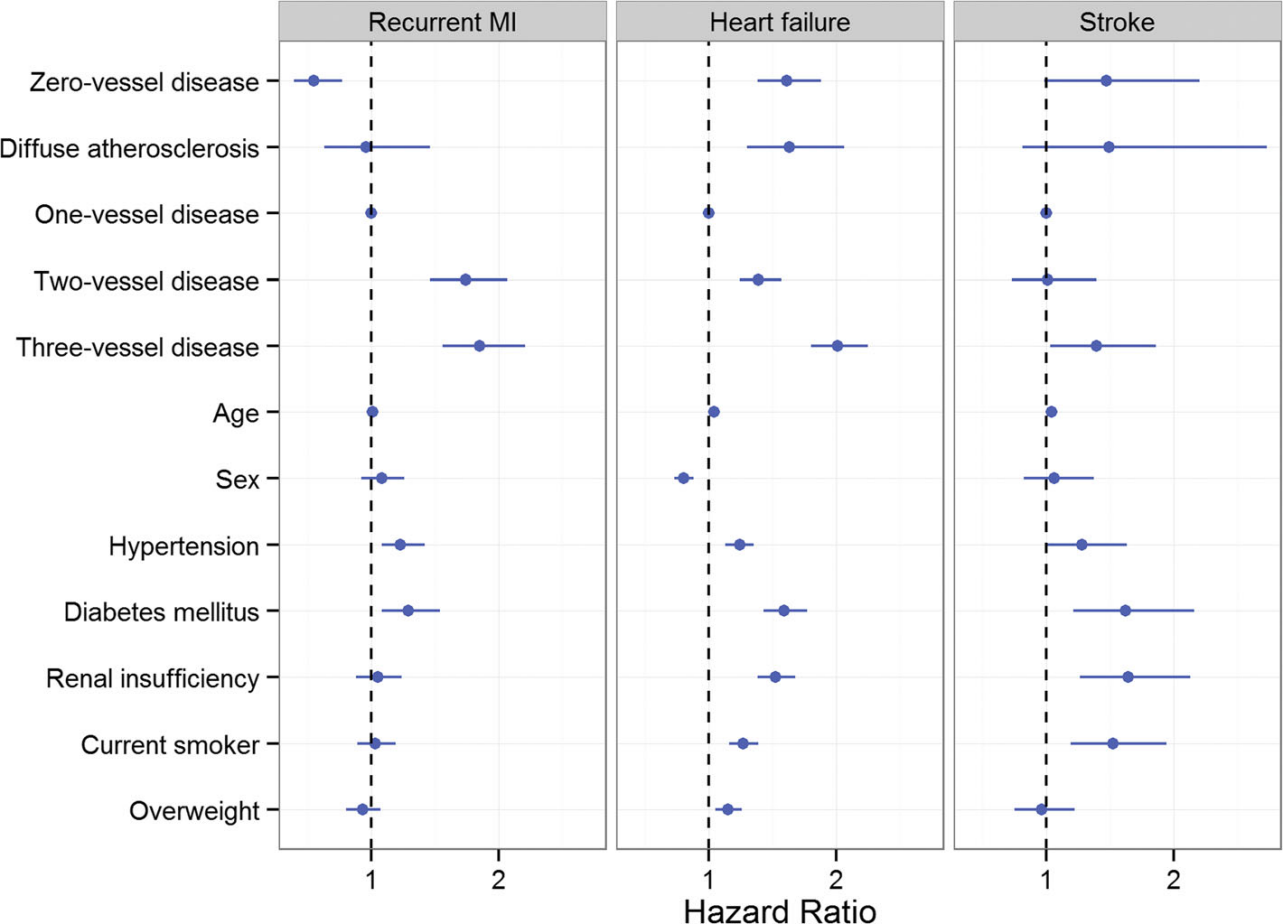
Adjusted hazard ratio of long-term recurrent myocardial infarction, heart failure, and stroke in patients with NSTEMI according to their coronary artery disease. Hazard ratio was adjusted for age, sex, hypertension, diabetes mellitus, renal insufficiency (eGFR < 6060 ml/min/1.73m^2^), current smoker status, and overweight (BMI ≥ 25)

Patients with 0VD had the lowest risk of recurrent MI in all groups after multi-factorial adjustment (Fig. 4), while patients with DA had a risk of recurrent MI that was higher than 0VD group (HR:1.7; 95% CI:1.05–2.89, *P* = 0.03), not significantly different from the 1VD (HR:0.91; 95% CI:0.60–1.39, *P* = 0.66), and lower than both the 2VD and 3VD groups. In patients with obstructive CAD, the risk of recurrent MI increased linearly with the severity of the disease.

#### Heart failure

**Fig. 5:**
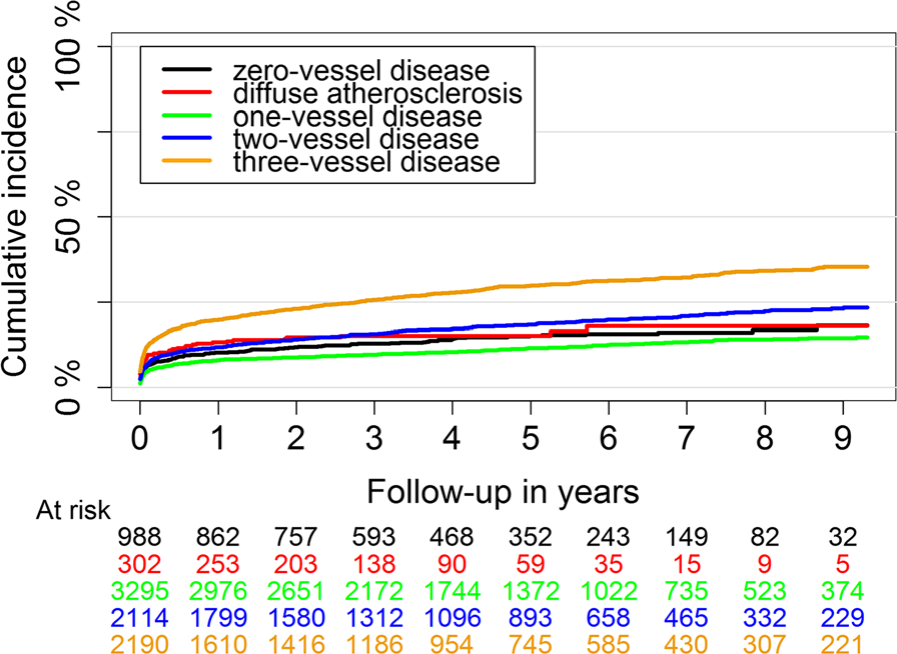
Long-term heart failure cumulative incidence in patients with first NSTEMI divided by their coronary artery atherosclerosis extent

One-year HF risk after first NSTEMI was substantial in patients with 0VD and DA (Table 2). Unadjusted cumulative incidence of HF was lowest in 1VD and highest in 3VD group (Fig. 5). After multi-factorial adjustment, the risk of HF was not significantly different between 0VD and DA groups. Both groups had higher HF risk compared with 1VD (Fig. 4), not statistically different compared with 2VD, and lower risk compared with the 3VD group (HR:0.80; 95% CI: 0.69–0.93, *P* = 0.004; and HR: 0.60; 95% CI: 0.44–0.83, *P* = 0.02), respectively.

#### Stroke

**Fig. 6:**
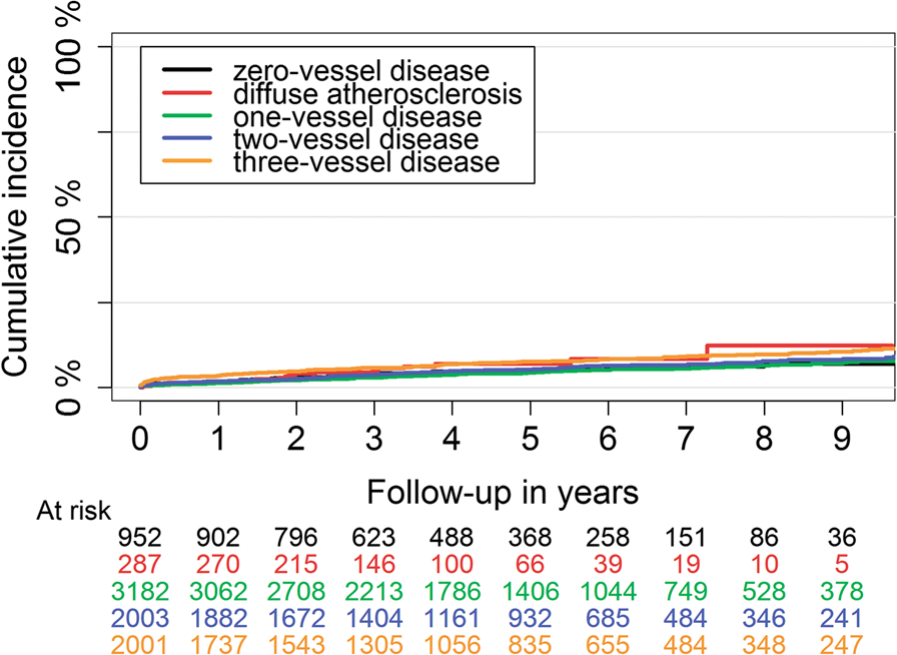
Long-term stroke cumulative incidence in patients with first NSTEMI divided by their coronary artery atherosclerosis extent

One-year stroke risk was highest in patients with 3VD and comparable in the other 4 groups (Table 2). Incidence of stroke was generally lower than other outcomes (Fig. 6). Adjusted risk of stroke in patients with 0VD compared with 1VD and 2VD were (HR:1.47; 95% CI: 0.98–2.20, *P* = 0.06) and (HR: 1.46; 95% CI: 0.95–2.23, *P* = 0.09), respectively. Stroke risk was similar in the 0VD compared with the DA and 3VD groups. Patients with DA had a similar stroke risk compared with all other groups.

### Additional analyses

#### Cardiovascular mortality

Considering only cardiovascular (CV) mortality and not all-cause mortality: for patients without DM, the CV mortality for 0VD group was higher compared to 1VD (HR:1.52; 95% CI: 1.19–1.94, *P* = <0.001), not different compared to DA and 2VD groups, and lower than 3VD group (HR: 0.69; 95% CI:0.50–0.96, *P* = 0.02). While patients in the DA group had a CV mortality which was higher than both 1VD and 2VD groups (HR: 1.82; 95% CI: 1.27–2.60, *P* = <0.001), (HR: 1.76; 95% CI: 0.91–1.82, *P* = 0.03), respectively, and not different compared to 3VD.

For patients with NSTEMI and DM, whose in the 0VD group had CV mortality which was not significantly different compared to both 1VD, and 2VD groups, but lower than CV mortality of both 3VD, and DA groups (HR: 0.06; 95% CI:0.007–0.47, *P* = 0.007). While patients in the DA group had higher CV mortality than 1VD (HR:2.88; 95% CI:1.29–6.43, *P* = 0.009), and not different compared to 2VD, and 3VD groups.

#### Patients with available EF measurement

This subgroup analysis consisted of 3986 patients. Multi-factorial adjustment including EF revealed that stroke hazard for 1VD group was significantly lower than all other groups. No other differences were observed for the other outcomes.

#### Sensitivity analysis considering recurrent MI only after 30 days

Recurrent MI risks from 30 to 365 days after NSTEMI were as follows: 0VD 0.7% (0.2–1.2%), DA 3.3% (1.3–5.5%), 1VD 2.4% (1.8–2.8%), 2VD 4.4% (3.6–5.3%) and 3VD 7.4% (6.3–8.4%). After adjustment for risk factors, 0VD continued to have the lowest risk of recurrent MI among all groups. Patients with DA had a not significantly different risk compared with the 1VD, 2VD, and 3VD groups.

## Discussion

Our study recruited 8889 patients with a follow-up duration of up to 13 years (median 4.5 years). It showed two important main findings: 1) NSTEMI patients with non-obstructive CAD (both 0VD and DA without significant stenosis) have a substantial risk of long-term adverse outcomes comparable to 1VD and 2VD patients; and 2) 0VD and DA groups are different in both risk profile and outcome.

This study showed that patients with 0VD were younger, mostly females, and had less co-morbidities than those with DA. This was also observed in another study [2]. An explanation of more females among both groups of non-obstructive CAD group could be that one half of females without significant stenosis have microvascular dysfunction [17].

The percentage of NSTEMI patients with non-obstructive coronary arteries increased gradually during the study to reach 18% in 2011, that might reflect the development of more sensitive troponin and the use of lower cut-off values in the definition of MI, and thus the detection of smaller injuries to the myocardium [18]. This means also that we are dealing with growing subgroup of patients who need more attention in our management.

### Mechanism of NSTEMI with non-obstructive CAD

There are several reported mechanisms of myocardial injury in these patients, like plaque disruption without significant stenosis on the angiogram proven with intra vascular ultrasound (IVUS) examination, [19] coronary artery spasm, [12, 20] microvascular disease, [21] or thrombophilias whether congenital or acquired [12]. Other possible non ischemic mechanisms including myocarditis which was observed in 7% of NSTEMI with non-obstructive CAD using cardiac MRI [3].

In our study 2.6% of 0VD and 5.0% of DA were treated with revascularization (either PCI or CABG). In the DA group, the explanation could be proven plaque rupture in non-obstructive lesion, and in the 0VD group, one of the explanations could be complications to the coronary angiography like perforation or dissection either with the diagnostic catheter or with optical coherence or IVUS wire. These complications might have led to revascularization in a patient without CAD.

### Long-term mortality after first NSTEMI

Our study illustrated that non-diabetic NSTEMI patients with 0VD had higher mortality risk compared with 1VD group. On the other hand, patients with DA had a 2-fold higher mortality than those with 1VD and similar mortality to those with 2VD.

Another study concluded that NSTEMI with normal coronary arteries had the same mortality risk as both atherosclerosis and low risk anatomy obstructive CAD [10] while other studies showed no significant difference in mortality in patients with and without obstructive CAD after adjustment for risk factors for IHD [1, 7, 22]. A recent study reported higher risk of one-year mortality in NSTEMI patients with non-obstructive compared with obstructive CAD, mostly driven by non-cardiac death [11]. These studies, however, did not divide obstructive CAD into subgroups. A possible explanation for our findings could be that as our results showed that patients with NSTEMI with non-obstructive CAD were less likely to receive double anti-platelet therapy and other recommended medical treatment after MI according to ESC guidelines, [23]. This was also shown in another study, where these patients were also more likely to discontinue double platelet inhibitors if these were started [1, 2]. Another explanation might be that many of these patients, especially those with 0VD, might have suffered a type II MI, and comprises a heterogeneous group with different background pathology leading to myocardial injury without CAD. Mortality risk of type II MI was reported to be comparable or higher than type I MI [24, 25].

### Recurrent MI

NSTEMI patients with 0VD had the lowest risk of recurrent MI among our five groups, and those with DA had the same risk as patients with 1VD. According to other studies, patients with non-obstructive CAD have lower risk of MI than obstructive CAD [2, 7]. However, these studies did not divide obstructive CAD into subgroups. Our findings might be explained by speculating that plaque burden might be comparable in the DA and 1VD groups; and both have higher plaque burden than the 0VD group. Previous studies using IVUS reported that plaque rupture is often not detected as significant stenosis by angiography, and is typically associated with eccentric and large plaque with positive remodeling [19, 26, 27]. A recent meta-analysis ([28] showed that shorter duration (<6 months) of double antiplatelet therapy, was associated with higher incidence of MI, less incidence of bleeding, and the same mortality and stent thrombosis rates. However, this study was in patients treated with PCI with second generation drug eluting stent, nevertheless, about 40% of patients were acute coronary syndrome patients. Although, our study population is different, but our results also indicated that a non-unreasonable anticipation is that some of the recurrent MI incidence in DA group is because of either not been given double anti-platelet therapy or shorter duration of treatment compared with obstructive CAD group.

### Heart failure

NSTEMI patients with both 0VD and DA had a higher risk of HF than the 1VD, same risk as the 2VD, and lower risk than the 3VD group in this study. Another study showed that the risk of HF in ACS patients with or without obstructive CAD was similar, [22] but that study did not differentiate sub groups and included only patients ≥75 years.

One explanation of our findings could be that some of the patients were admitted with acute HF with symptoms, signs and troponin elevation that resembled MI. It is well known that HF can cause myocardial injury resembling MI [29]. However, we tried to restrict that possibility by excluding patients known with HF. Another explanation may be that especially patients with 0VD could have a myocardial disease in early stage which developed afterward to a manifest HF.

### Stroke

Patients with NSTEMI with non-obstructive CAD had a similar risk of stroke compared with those with obstructive CAD. This confirms the findings of other studies [1, 2].

## Study strength and limitations

The strengths include the large number of unselected patients with NSTEMI. Patients from both genders and all age groups were included, which helps generalization of our results.

However, our study has some limitations; patients with MI type II might have been included if the primary discharge diagnosis was NSTEMI, and it seems to be reasonable to assume that this was more applicable to 0VD and DA groups. These considerations, however, do not change that, according to our data, these two groups remain at high risk of adverse events. In angiography database the definitions of 0VD and DA were subject for inter- and intra-hospital different interpretations, thus some of the patients with DA might have been coded as 0VD. However, this misclassification would lead to minimize the differences between 0VD and DA groups, thus the real differences between these groups in outcome may be larger than that reported in our article. A core-lab for coronary angiography assessment was not used, thus inter-operator variation might exist. Lastly, only patients who underwent coronary angiography were included which make generalization of the results limited. The cut-off values and Troponin assays has been changed during the study years, which might led to that the MI size detected in the later years are smaller than that at the earlier years of the study.

## Conclusion

Patients with NSTEMI with normal coronary arteries or atherosclerosis without significant stenosis have substantial risk of future cardiovascular events. Both patients with 0VD without diabetes and patients with DA have higher risk of mortality and heart failure compared with patients with 1VD; and patients with DA have a similar risk of recurrent MI compared with those with 1VD. Moreover, patients with 0VD have favorable risk profile, lower mortality (if patients were diabetic) and lower recurrent MI risk than patients with diffuse atherosclerosis. These findings call for considering these two subgroups of NSTEMI separately in future research and urge for further investigations to explore the best management and follow-up plans for each of these two subgroups.

## Additional file

Additional file 1: Table S1. Hazard ratio of outcome events in NSTEMI patients divided by coronary artery disease. (DOCX 15 kb)

## Abbreviations

0VD: zero-vessel disease
1VD: one-vessel disease
2VD: two-vessel disease
3VD: three-vessel disease
CAD: Coronary artery disease
DA: Diffuse atherosclerosis without significant stenosis
DM: Diabetes mellitus
HF: Heart failure
NSTEMI: Non-ST-segment elevation myocardial infarction
