# Supplementary material for: Long-term prognosis of patients with non-ST-segment elevation myocardial infarction according to coronary arteries atherosclerosis extent on coronary angiography: a historical cohort study
Source: BMC Cardiovasc Disord. 2017 Nov 16;17:279. doi: 10.1186/s12872-017-0710-3 (PMC5689183; doi:10.1186/s12872-017-0710-3)
Supplement: Additional file 1: Table S1. — Hazard ratio of outcome events in NSTEMI patients divided by coronary artery disease. (DOCX 15 kb) [file 12872_2017_710_MOESM1_ESM.docx]

**Table S1** Hazard ratio of outcome events in NSTEMI patients divided by coronary artery disease

|  | **Mortality without DM** | **Mortality with DM** | **Recurrent MI** | **Heart failure** | **Stroke** |
| --- | --- | --- | --- | --- | --- |
| **0VD** | 1.59 (1.21-2.02) ** | 0.40 (0.14-1.13) | 0.55 (0.39-0.77) ** | 1.61 (1.39-1.88) ** | 1.47 (0.98-2.20) |
| **DA** | 1.93 (1.31-2.83) ** | 2.50 (1.28-4.90)* | 0.96 (0.63-1.46) | 1.63 (1.30-2.06) ** | 1.49 (0.81-2.73) |
| **1VD** | 1.0 | 1.0 | 1.0 | 1.0 | 1.0 |
| **2VD** | 1.33 (1.08-1.63)* | 1.54 (1.02-2.31)* | 1.74 (1.46-2.07) ** | 1.39 (1.24-1.57) ** | 1.01 (0.73-1.39) |
| **3VD** | 1.92 (1.95-2.31) ** | 1.53 (1.06-2.21) ** | 1.85 (1.56-2.21) ** | 2.01 (1.80-2.25) ** | 1.39 (1.03-1.86)* |

* P<0.05, ** P<0.001. 1VD group was used as reference group.

Abbreviations: 0VD zero-vessel disease, DA diffuse atherosclerosis, 1VD one-vessel disease, 2VD two-vessel disease, 3VD three-vessel disease, DM diabetes mellitus.
